# Supplementary material for: Flotillin-mediated stabilization of unfolded proteins in bacterial membrane microdomains
Source: Nat Commun. 2024 Jul 3;15:5583. doi: 10.1038/s41467-024-49951-1 (PMC11222466; doi:10.1038/s41467-024-49951-1)
Supplement: Supplementary file 1 — Supplementary Information [file 41467_2024_49951_MOESM1_ESM.pdf]

Supplementary Information

Supplementary Figure 1

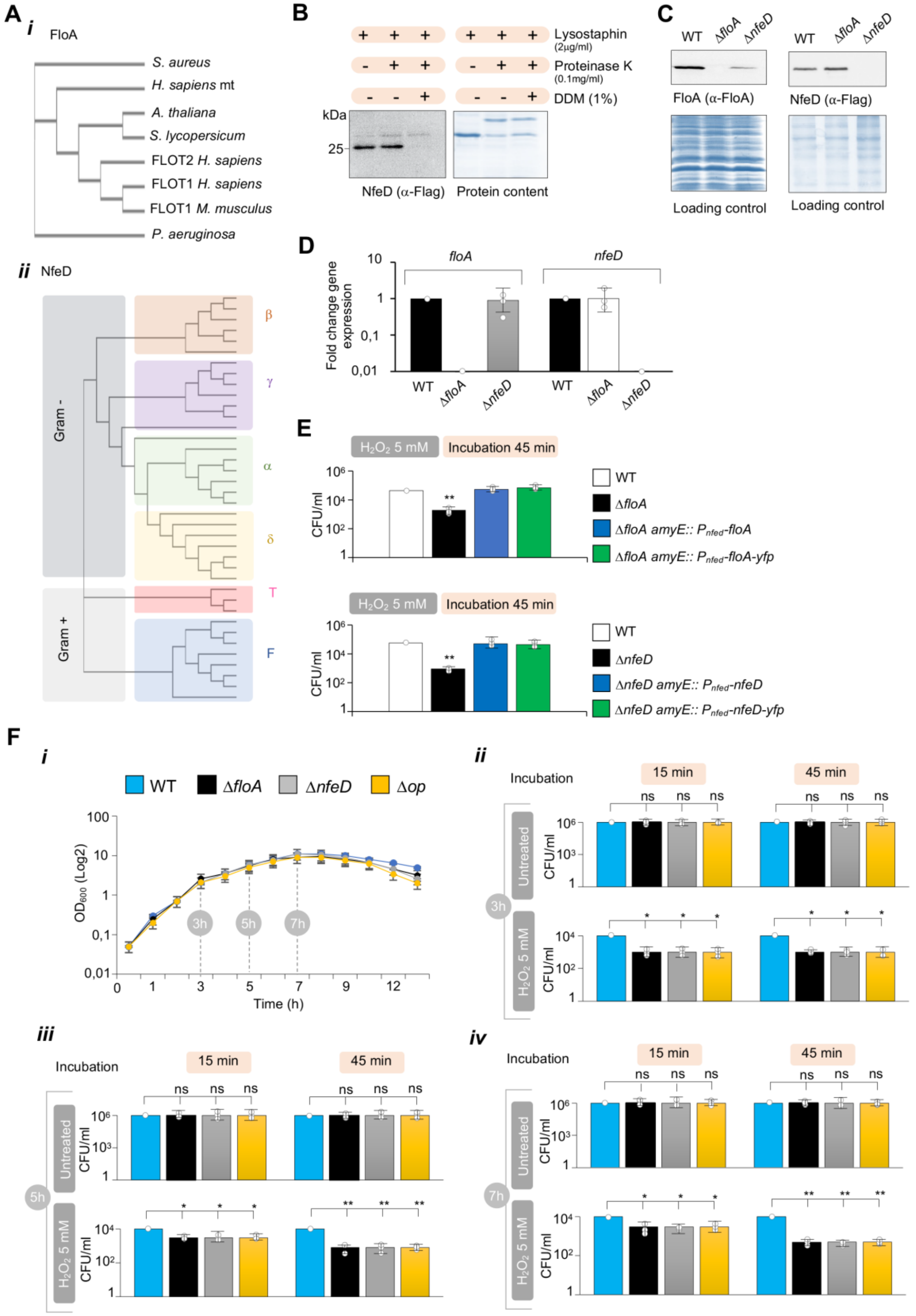

Supplementary Figure 1. Generation of Δ*floA* and Δ*nfeD* mutants. A) (i) Schematic phylogeny of different flotillin proteins**.** From top to bottom, *Staphylococcus aureus* FloA, *Homo sapiens* prohibitin from mitochondria, *Arabidopsis thaliana* flotillin, *Solanum licopersiucum* flotillin, flotillin FLOT2 from *Homo sapiens*, flotillin FLOT1 from *Homo sapiens*, FLOT1 from *Mus musculus* and flotillin from *Pseudomonas aeruginosa*. Flotillin and flotillin-related proteins conserved many of their structural features along the phylogenic tree. (ii) Phylogeny of *nfeD* in bacterial genomes; β, γ, α and δ proteobacteria as well as thermococci (T) and firmicutes (F). NfeD is present in almost all bacterial species as a flotillin partner protein. NfeD is exclusively present in prokaryotes and is not present in eukaryotes. **B)** Topology of NfeD facing the bacterial cytoplasm. NfeD-FLAG produced in *S. aureus*. Cells were partially lysed with lysostaphin and treated with proteinase K to digest the proteins. Immunodetection of the FLAG tag was not possible when the membrane was permeabilized with DDM before the proteinase treatment (lane 3), suggesting that NfeD C-terminus faces the cytoplasm. The coomassie stained gel served as loading control. **C)** Immunodetection of FloA or NfeD in Δ*nfeD* and Δ*floA* MRSA mutants. **D)** qRT-PCR analysis of *floA* and *nfeD* gene expression levels in TSB cultures of different *S. aureus* strains. Data are shown as mean ± SD of three independent experiments (n = 3). **E)** Susceptibility to H_2O_2 5 mM (at 15 min or 45 min incubation period) of Δ*floA* or Δ*nfeD* mutants; mutants complemented with a WT copy of the gene (Δ*floA floA* or Δ*nfeD nfeD*) and mutants complemented with a YFP-labeled version of the protein (Δ*floA floA-yfp* or Δ*nfeD nfeD-yfp*) measured as CFU count. The mutants showed severe susceptibility to H_2O_2 treatment whereas the complemented strain restored the CFU count to levels comparable to WT. Statistical differences were measured by one-way ANOVA with Tukey's test for multiple comparison, $**p<0.01$. Data are shown as mean ± SD of three independent experiments (n = 3). **F)** (i) Growth curve of WT and mutants (Δ*floA*, Δ*nfeD* and Δoperon) in TSB medium. Cultures were incubated for 12h at 37°C with 200 rpm agitation. Samples were taken at 3h (ii), 5h (iii) and 7h (iv) incubation periods to assay H_2O_2 susceptibility (H_2O_2 5 mM at 15 min or 45 min incubation period). Statistical differences were measured by one-way ANOVA with Tukey's test for multiple comparison, $*p<0.05$, $**p<0.01$. Data are shown as mean ± SD of three independent experiments (n = 3).

Supplementary Figure 2

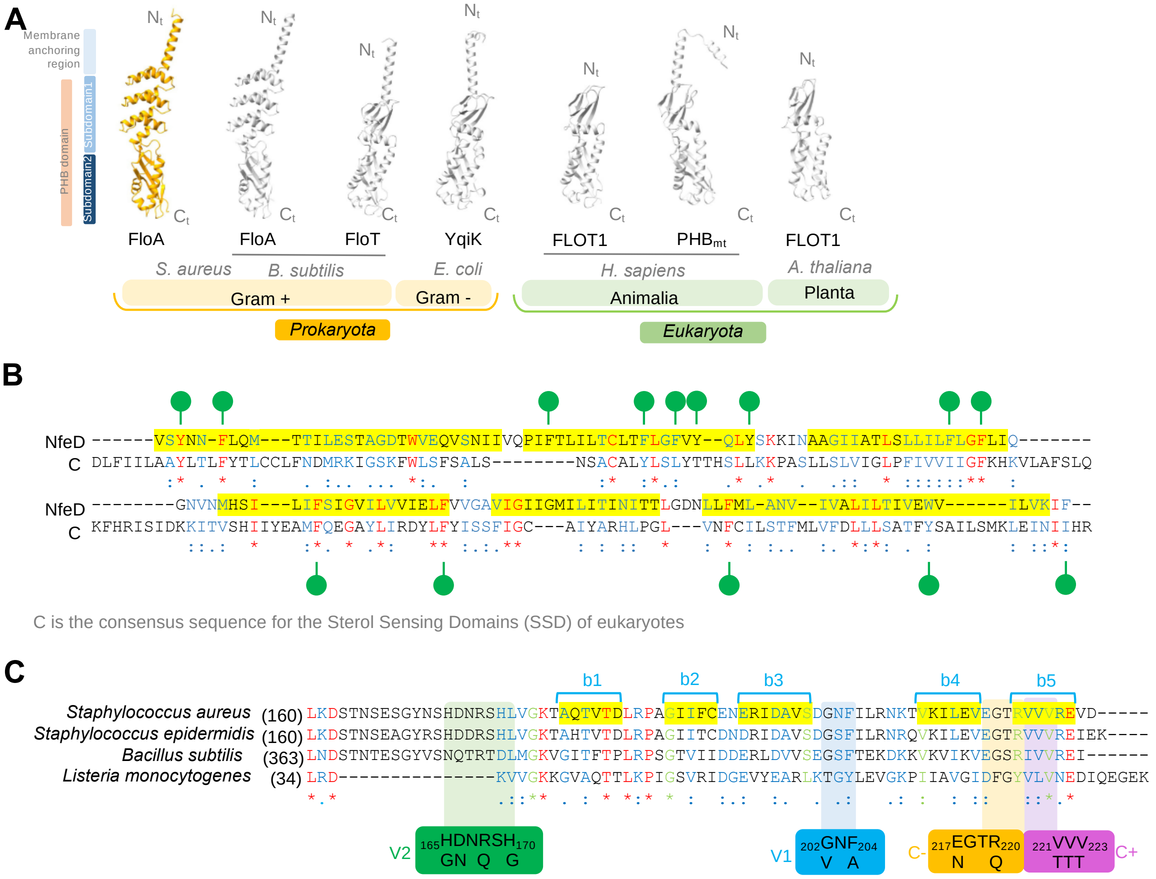

Supplementary Figure 2. Interaction of NfeD with FMM lipids. A) Alphafold2 prediction of the organizational structure of PHB domains from flotillins of distinct organisms. The subdomain 1 of FloA in *S. aureus* is similar to that of FloA in *B. subtilis* but it differs from the PHB subdomain 1 of other flotillins. In contrast, subdomain 2 of PHB is highly conserved in all flotillin and flotillin-related proteins from prokaryotes and eukaryotes. **B)** Amino acid sequence alignment of the NfeD transmembrane region and the eukaryotic SSD consensus sequence (C). The transmembrane regions are highlighted in yellow. The amino acids highlighted in red or blue with one or two dots indicate identical residues in all sequences, conserved and semi-conserved substitutions, respectively. The aromatic residues that were altered by site-directed mutagenesis in the NfeD$_{SSDL}$ variant are marked with a green flag. **C)** Amino acid sequence alignment of the NfeD OBL from different Gram-positive bacterial species. The β-sheets are marked in yellow; the amino acids highlighted in red (and red asterisk) indicate identical residues in all sequences. The amino acids highlighted in green (and green asterisk) indicate conserved residues important for ligand binding.

The amino acids highlighted in blue with one or two dots indicate conserved and semi-conserved substitutions, respectively. The regions mutagenized in the variants that were tested using a B2H assay are highlighted with their respective colors. In the V1 region, we replaced the conserved aromatic residues with non-aromatic residues. In the V2 region, we also replaced charged residues with non-charged residues. C+ is a positive control. In this region, we replaced hydrophobic amino acids with hydrophilic amino acids, which likely affected the conformation of the barrel. C- is a negative control. Changes in this loop should not affect the structure of the barrel and were predicted to be distant from other interacting regions. Therefore, variations in these regions should not interfere with the interactions.

Supplementary Figure 3

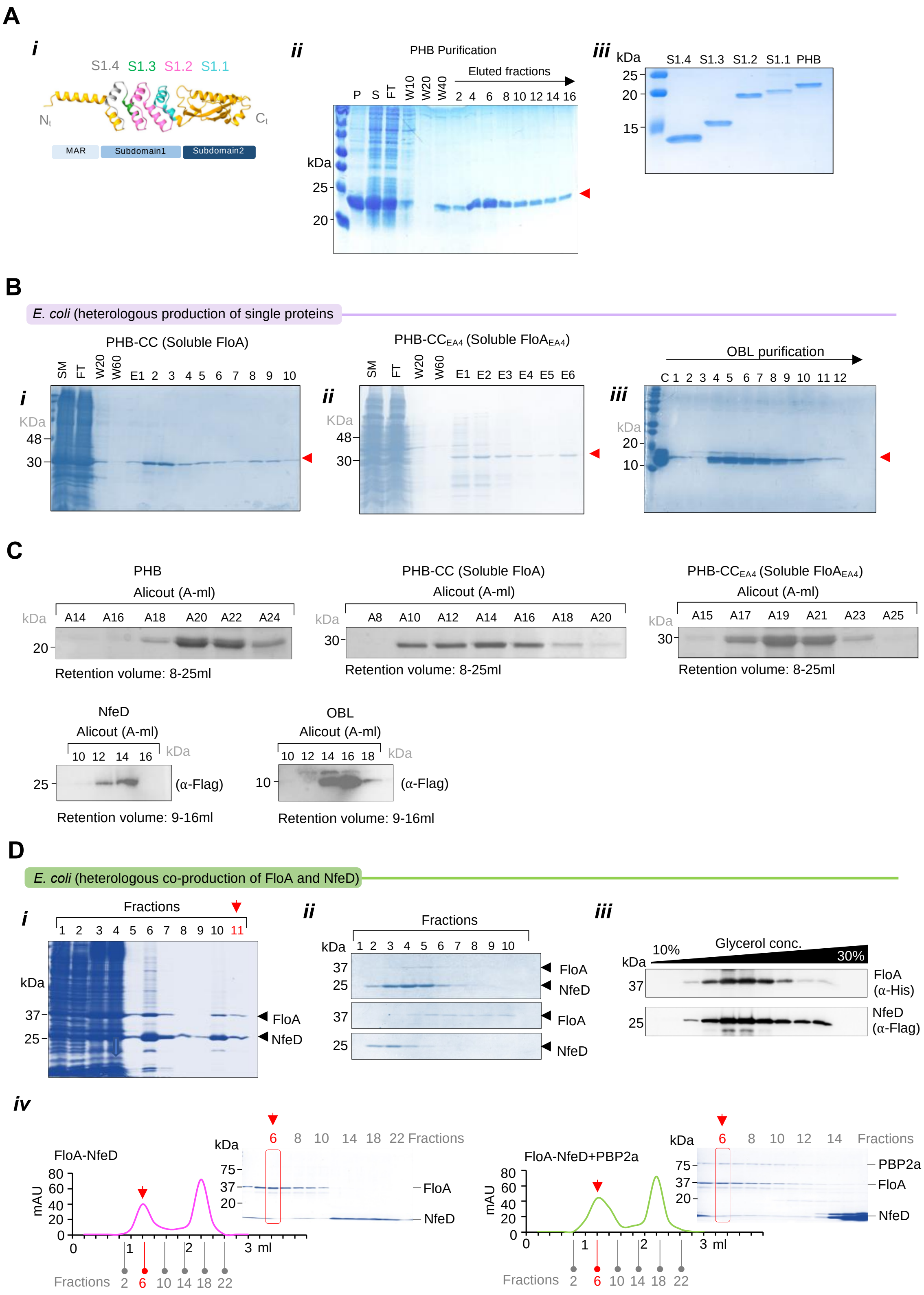

Supplementary Figure 3. Interaction of FloA with FMM lipids A) (*i*) Schematic representation of the deletions generated in the S1-4 variants of the PHB. α-helices of the PHB subdomain 1 were systematically deleted in the S1-4 variants. The α-helices deleted in each variants are colored. (*ii*) PHB purification steps. P is the pellet, S is the supernatant. FT is the flow through. Washing steps with 10, 20 or 40 mM imidazole are shown as W10, W20 and W40, respectively. Fractions 2-16 represent the elution fractions. The red arrow indicates the band of the PHB domain. (*iii*) SDS-PAGE of the different purified PHB variants (S1.1-4) and the WT PHB domain. **B)** Purification of FloA and FloA-NfeD. SDS-PAGE shows the purification steps of the distinct FloA variants (i and ii) and the OBL from NfeD (iii). SM = soluble membrane; FT = flow through; W20 = washing buffer imidazole 20mM; W60 = washing buffer imizadole 60mM; E = elution steps. C is a crude extract. **C)** Upper panels, detection of PHB (left panel), PHB-CC (centre panel) and PHC-CC_{EA4} (right panel) signal in the aliquots of the SEC profiles presented in Figure 2F. Bottom panels; immunodetection of NfeD (left panel) and OB domain (right panel) in the aliquots of the SEC profiles presented in Figure 2G. Aliquots (A-ml) represent the retention volume in ml units. **D)** Heterologous co-production and purification of FloA and NfeD in the same *E. coli* strain. (i) SDS-PAGE showing the purification steps of FloA and NfeD in the same protein extracts (marked with black arrows). Red arrow marks the fraction that was analyzed by Cryo-EM. (ii) Glycerol gradient analysis of FloA-NfeD purification fractions. FloA and NfeD co-migrated to the same gradient fractions, whereas single purified FloA or NfeD proteins migrate to different fractions. This is indicative that FloA and NfeD interact when co-produced in the same strain. (iii) Immunodetection of FloA and NfeD in the same protein fractions of the glycerol gradient, which is indicative of FloA-NfeD interaction, as both are present in different fractions of the glycerol gradient. (iv) SEC profiles for the isolation of 2xFloA-NfeD (left panel) and 2xFloA-NfeD+PBP2a (right panel) using a Superdex200 5/150 column (GE). Fraction 6 highlighted in red was selected for Cryo-EM analyses. In the right panels, protein samples from the SEC fraction were resolved by SDS-PAGE. Fraction 6 highlighted in red was selected for Cryo-EM analyses.

Supplementary Figure 4

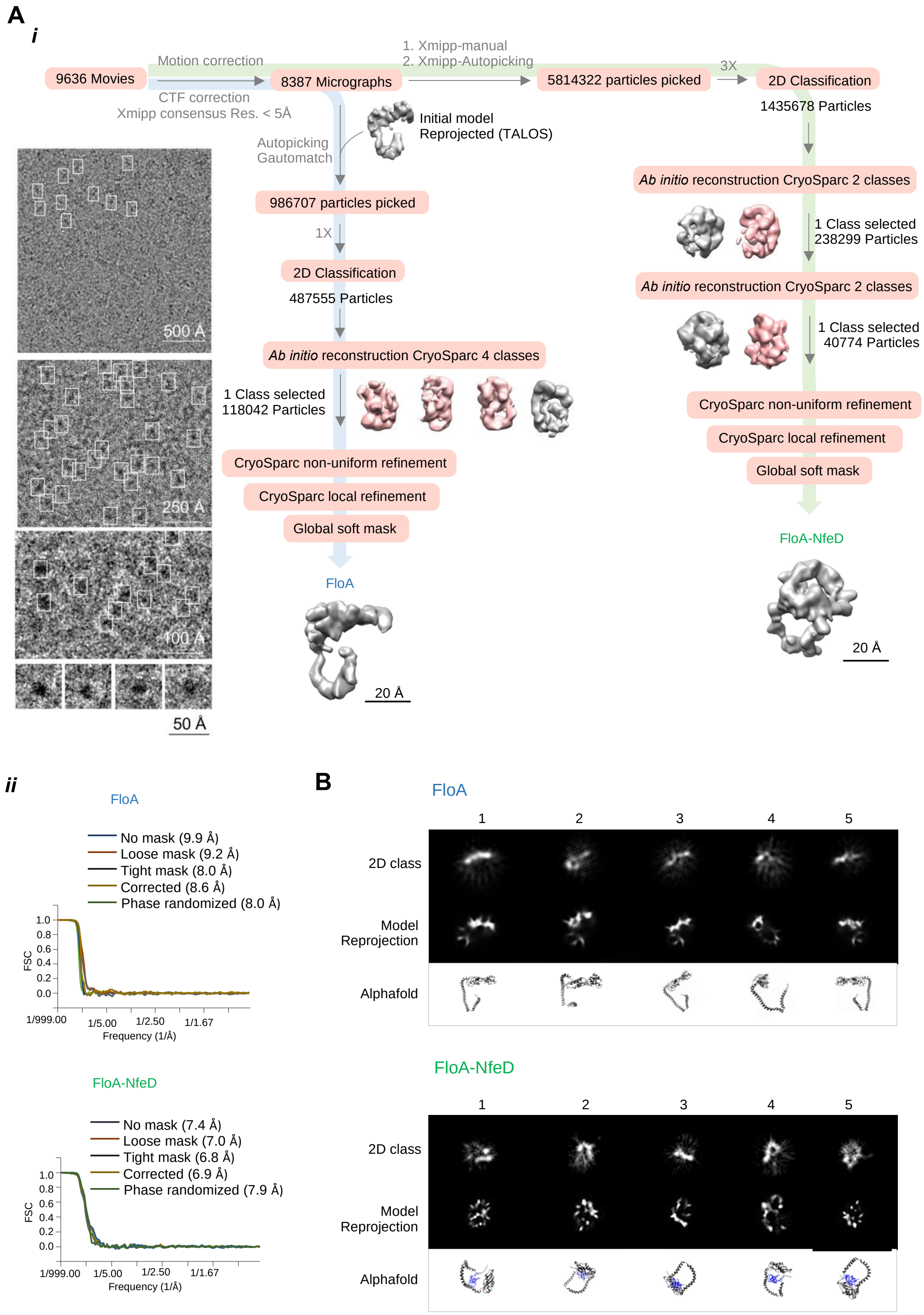

Supplementary Figure 4. Workflow of Cryo-EM image processing. A) Schematic representation of the data collection and image processing, which is described in the methods section. The initial pre-processing of the movies was performed on-the-fly during data collection using CryoSPARC Live. A representative cryo-EM micrograph of the sample is shown. White squares highlight individual particles. To facilitate particle visualization, we present sequential micrographs featuring magnified areas where particles are delineated by white squares. The bottom panels provide detailed views of four selected particles at high magnification. Dose-weighted micrographs were imported and processed using Scipion. The final EM map of FloA monomer (blue line) was obtained from C1 3D masked local refinement of 118042 particles at the resolution of 8.0 Å (phase randomized). The left panel shows the Fourier Shell Correlation FSC [0.143] curves. The final FloA-NfeD EM model (green line) was obtained from C1 3D masked local refinement of 40774 particles at the resolution of 7.9 Å (phase randomized). The left panel shows the Fourier Shell Correlation FSC [0.143] curves. **B)** 2D class averages obtained from 2D classification of FloA (upper panel) and FloA-NfeD (bottom panel) monomers. The different 2D classes are presented in the top row. The center row are views of FloA (upper panel) or FloA-NfeD (bottom panel) 2D reprojections of the obtained cryo-EM map whereas the atomic model (Alphafold) is presented in the bottom row. 2D class averages concurring with the 2D reprojections of the cryo-EM map shows the accuracy of data processing.

Supplementary Figure 5

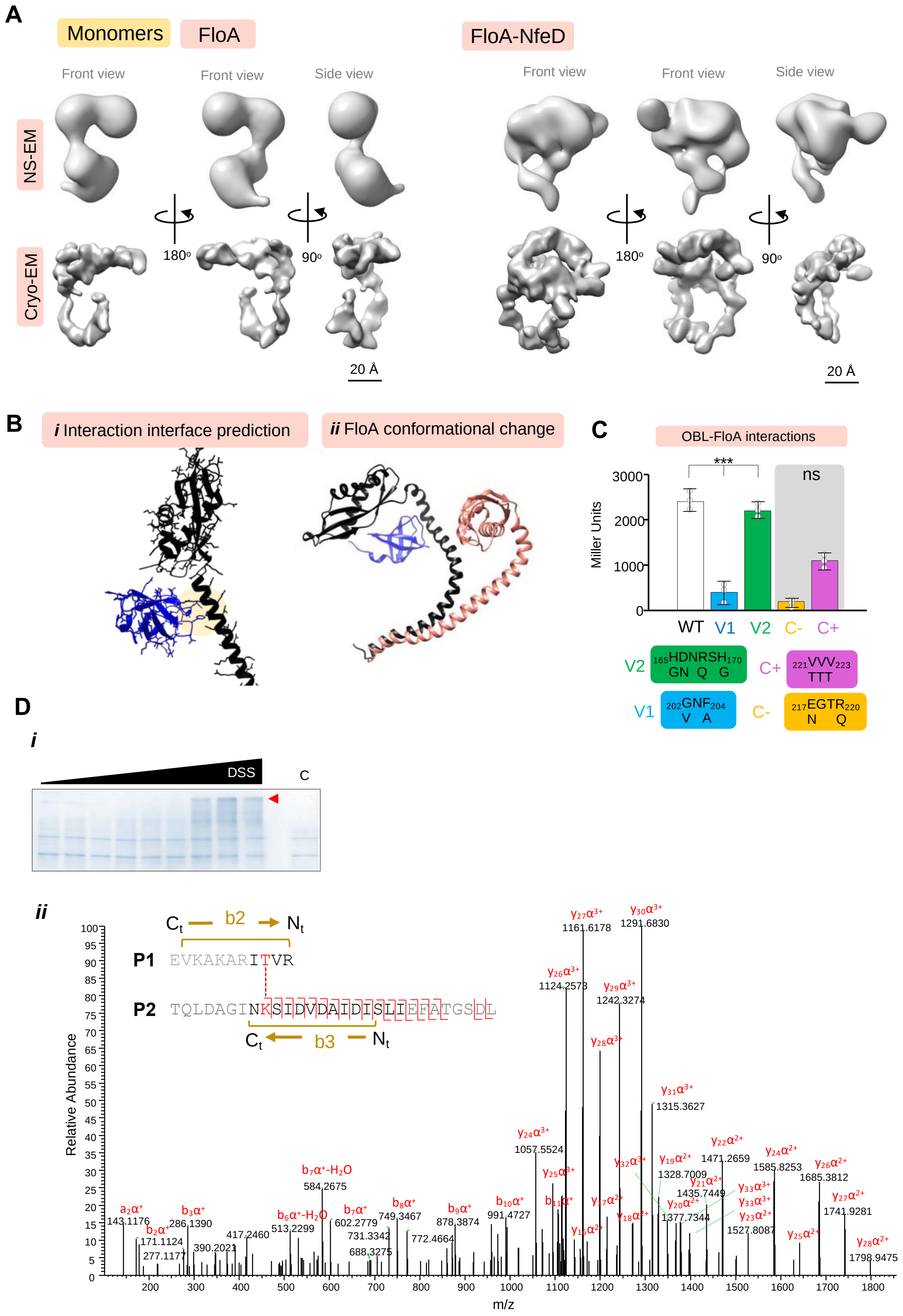

Supplementary Figure 5. Interaction of FloA and NfeD. A) Different views of 3D maps of FloA (left panel) and FloA-NfeD (right panel) monomers obtained by NS-EM (upper row) and cryo-EM (bottom row). Consistently, comparable particles in shape and size were obtained using two independent sample preparation and data processing (NS-EM and cryo-EM), which is indicative of the accuracy of cryo-EM data processing. **B)** (*i*) Alphafold2 AF2 Multimer prediction of FloA (colored in black) and OBL (colored in blue) interaction interface (highlighted in yellow). (*ii*) Superimposition of the FloA (orange) and FloA-NfeD structures (black) to denote the FloA conformational changes caused by OBL binding. **C)** CC-OBL interaction efficiency of the WT and OBL variants V1, V2, C+ and C- using a B2H assay (β-gal activity). Results were examined by one-way ANOVA with Tukey test for multiple comparisons; ***$p<0.001$. Data are shown as mean ± SD of three independent experiments (n = 3). **D)** (*i*) SDS-PAGE showing FloA oligomerization at increasing concentrations of DSS. Increasing the concentration of DSS added to the sample promoted the occurrence of FloA oligomers in the sample. The red arrow indicates the band corresponding to a FloA oligomer what was analyzed by mass spectrometry (MS). (*ii*) Experimental MS2 spectrum acquired after fragmentation of a 4+ precursor ion at m/z = 1118.832 during LC-MS/MS analysis of tryptic peptides derived from the DSS-crosslinked FloA sample. Mayor product ions are annotated in red. The identified peptide sequence identified is shown in the upper left corner. The peptidic fragments detected by MS are defined by red wedges and the DSS crosslink in red dashed line.

Supplementary Figure 6

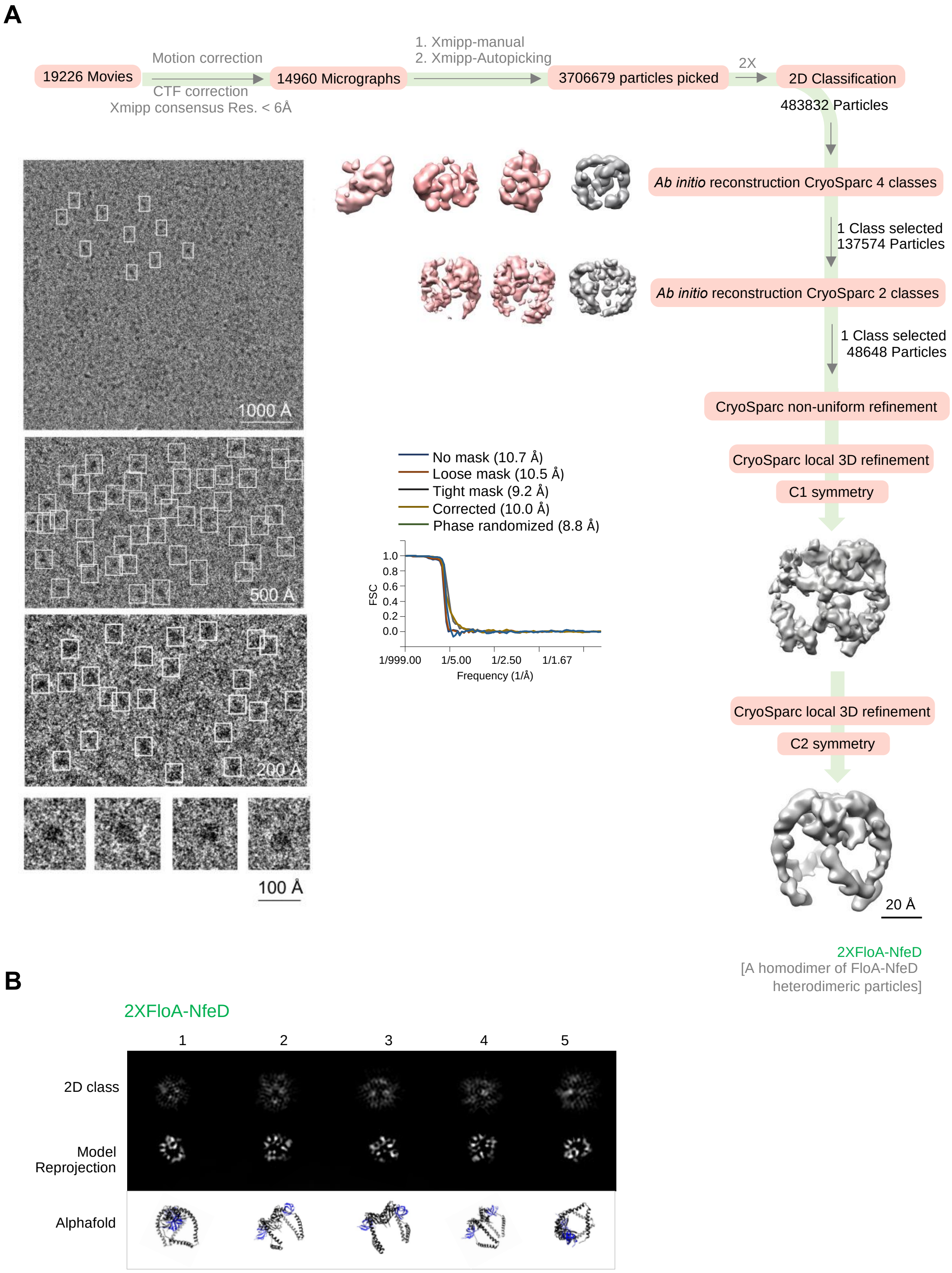

Supplementary Figure 6. Workflow of Cryo-EM image processing. A) Schematic representation of the data collection and image processing of FloA-NfeD dimers. The initial pre-processing of the movies was performed on-the-fly during data collection using CryoSPARC Live. A representative cryo-EM micrograph of the sample is shown. White squares highlight individual particles. White squares highlight individual particles. To facilitate particle visualization, we present sequential micrographs featuring magnified areas where particles are delineated by white squares. The bottom panels provide detailed views of four selected particles at high magnification. Dose-weighted micrographs were imported and processed using Scipion. The final EM map of FloA-NfeD dimer (2XFloA-NfeD) was obtained from C2 3D masked local refinement of 48648 particles at the resolution of 8.8 Å (phase randomized). The left panel shows the Fourier Shell Correlation FSC (0.143) curves. **B)** 2D class averages obtained from 2D classification of FloA-NfeD dimers. The different 2D classes are presented in the top row. The center row are views of the 2D reprojections of the obtained cryo-EM map whereas the atomic model (Aphafold) is presented in the bottom row. 2D class averages concurring with the 2D reprojections of the cryo-EM map shows the accuracy of data processing.

Supplementary Figure 7

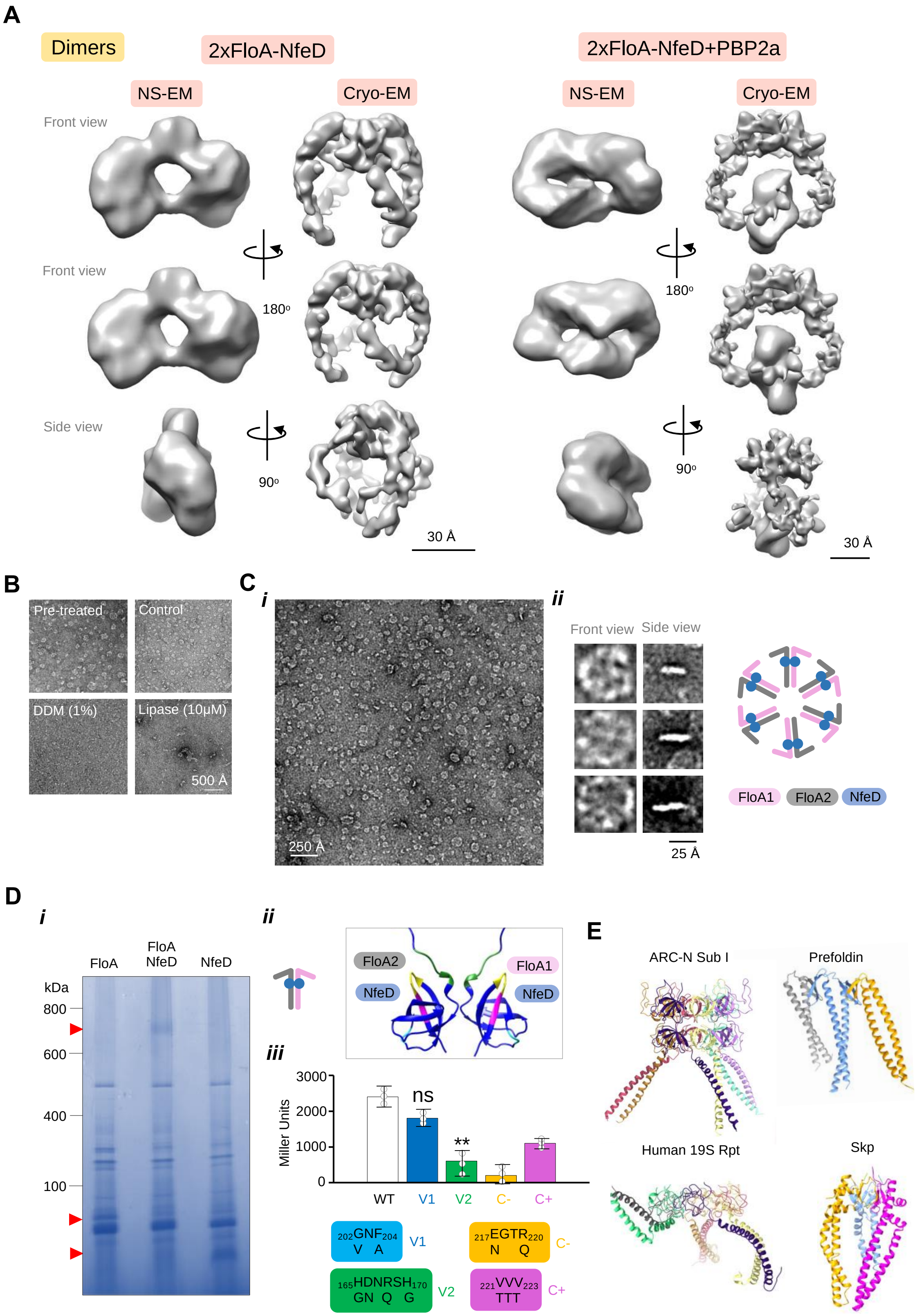

Supplementary Figure 7. Analysis of FloA complex assembly and disassembly. A) Different views of 3D maps of FloA-NfeD dimers in their apo state (left panel) or bound to unfolded PBP2a (right panel). Each panel compares the data obtained by NS-EM (left column) and cryo-EM (right column). Consistently, comparable particles in shape and size were obtained using two independent sample preparation and data processing (NS-EM and cryo-EM). The dimeric particles were comparable in shape and size using two independent sample preparation and data processing methodologies (NS-EM and cryo-EM). **B)** FloA oligomer disassembled upon treatment with DDM (0.1%) or lipase (10 μM); pre-treated sample, and a sample treated with buffer only, showing the oligomeric arrangements whereas the oligomeric structures disappeared in treated samples. **C)** (*i*) Negative staining EM micrographs of a FloA oligomeric sample. The multimeric assemblies were visible by EM but disaggregated in the absence of membrane lipid; dimers and assemblies of dimers were observed. (*ii*) Detailed micrographs of several multimeric particles at different views. It is possible to visualize the formation of ring-shaped multimeric assembles. An schematic reconstruction is shown to the right. One FloA monomer is labeled in pink and the other FloA monomer in grey. The OBL of NfeD is labeled in blue. **D)** (*i*) BN-PAGE of *E. coli* extracts overproducing the staphylococcal FloA and/or NfeD. Each one of the strains producing the heterologous FloA or NfeD showed an extra band below 100 kDa (marked on the left with an arrowhead). In contrast, the strain overproducing both FloA and NfeD showed an extra band at ~700 kDa, suggesting that FloA and NfeD overproduction is important for oligomerization. (*ii*) Representation of AF2 predictions of two FloA dimers. One FloA monomer from one dimer is labeled in pink and the other FloA monomer from the dimer is labeled in grey. The OBL of NfeD is labeled in blue. The dimer-to-dimer interaction may occur at the part of the tentacle bound to the OBL domain. The OBL sites that were mutagenized to validate dimer-to-dimer interaction are colored. (*iii*) Dimer-to-dimer interaction efficiency of the different OBL variants (V1, V2, C+ and C-) using a B2H assay (β-gal activity). Results were examined by one-way ANOVA with Tukey test for multiple comparisons; $^{**}p<0.01$. Data are shown as mean ± SD of three independent experiments (n = 3). **E)** Structural conformation of different CC-OB-containing chaperones. ARC-N is the subcomplex I in archaea and eukaryotic 19S Rpt, prefoldin and the periplasmic chaperone Skp. The chaperones show a region constituted by tentacles in which a coiled-coil region is linked to an OB-fold domain. In prefoldin and Skp, the OB region is reduced to two antiparallel β-sheets.

Supplementary Figure 8

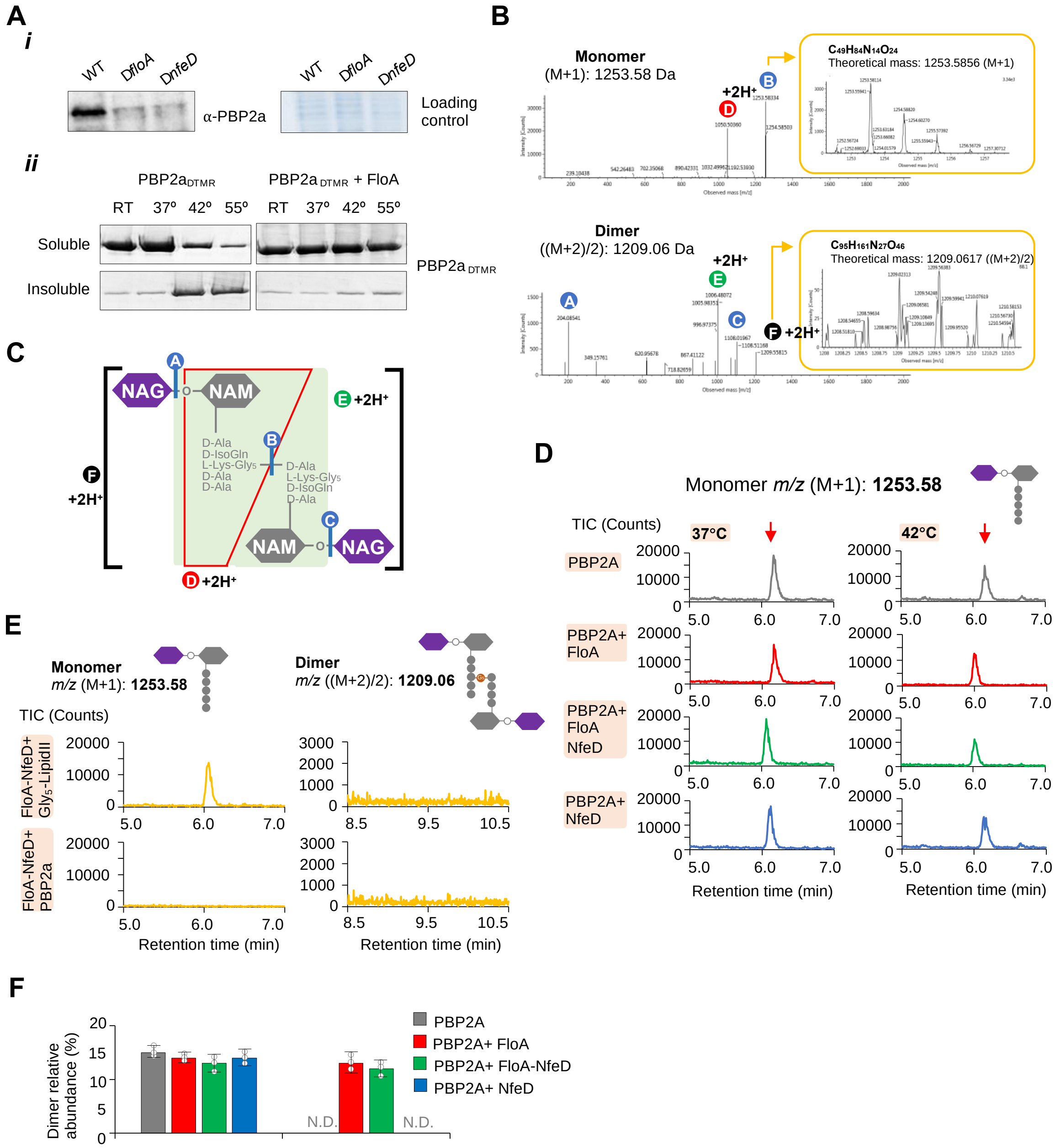

Supplementary Figure 8. Functional analyses of FMM client proteins in the presence and absence of FloA. A) (i) Immunodetection of soluble, functionally active PBP2a in the membrane fraction of different MRSA strains. PBP2a was detected in WT strain and contrasted with the poor PBP2a detection in Δ*floA* and Δ*nfeD* mutants. The coomassie stained gel served as loading control. (ii) Thermal aggregation assay of the PBP2a$_{\Delta TMR}$ variant that lacked the transmembrane region conducted at various temperatures. Similar to the behavior of PBP2a, PBP2a$_{\Delta TMR}$ showed a strong

onset of insolubility at 42°C. The addition of FloA to the assay reduced PBP2a$_{\Delta TMR}$ insolubility. **B)** LC/MS/MS fragmentation patterns of monomer (top panel) and dimer (bottom panel) muropeptide obtained from the *in vitro* reactions. The high-resolution mass spectra of the monomer and dimer muropeptide are close to the theoretical mass spectra, which are represented in a right panel with a yellow frame. **C)** Chemical structure of the dimer muropeptide and fragmentation patterns. **D)** LC-MS extracted ion chromatogram using *S. aureus* PBP2a and Gly5-Lipid II in different combinations with FloA and/or NfeD at 37ºC and 42ºC. The resulting reactions produced, in all cases, the monomeric muropeptide, which is shown in this panel. However, the production of the dimeric muropeptide at 42°C only occurs in the presence of FloA into the reaction (Fig. 6). The following ions were extracted from each chromatogram: Monomer: 1,253.5856 (M+1); and Dimer: 1,209.0617 ([M+2]/2). PBP2^{S398G} (PBP2 variant containing an inactive transpeptidase domain) was used to polymerize Gly$_5$-Lipid II as a substrate for PBP2a. **E)** Top row, LC/MS extracted ion chromatogram controls of *S. aureus* FloA-NfeD with Gly$_5$-Lipid II and lacking PBP2a. No transpeptidase activity was detected (dimer muropeptide). Bottom row, LC/MS extracted ion chromatogram controls of *S. aureus* FloA-NfeD with PBP2a lacking Gly$_5$-Lipid II showing no resulting muropeptide species. PBP2^{S398G} (PBP2 variant containing an inactive transpeptidase domain) was used to polymerize Gly$_5$-Lipid II as a substrate for PBP2a in both reactions. **F)** Dimer relative abundance between conditions. Data are shown as mean ± SD of three independent experiments (n = 3). n.d. is not detected. PBP2a incubated at different temperatures in the presence or absence of FloA. Integrated peaks from cross-linked muropeptides regarding the total number of muropeptides determined using the standard equation (sum of dimer integrated area/2 divided by the total integrated area of all muropeptide species observed).

Supplementary Figure 9

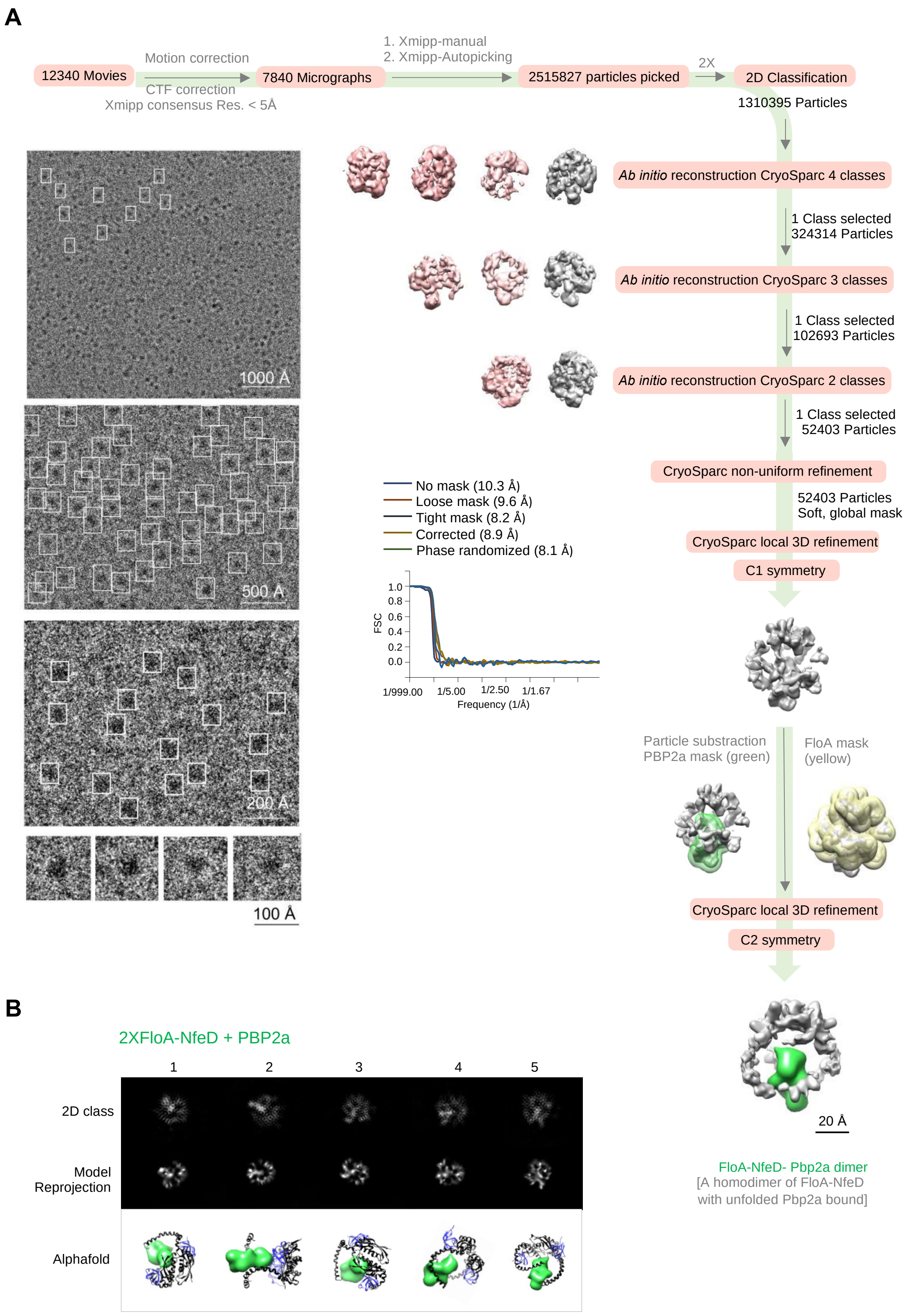

Supplementary Figure 9. Workflow of Cryo-EM image processing. Schematic representation of the data collection and image processing of FloA-NfeD dimers bound to unfolded PBP2a. The initial pre-processing of the movies was performed on-the-fly during data collection using CryoSPARC Live. A representative cryo-EM micrograph of the sample is shown. White squares highlight individual particles. To facilitate particle visualization, we present sequential micrographs featuring magnified areas where particles are delineated by white squares. The bottom panels provide detailed views of four selected particles at high magnification. Dose-weighted micrographs were imported and processed using Scipion. The final EM map of (2XFloA-NfeD + PBP2a) FloA-NfeD dimer-Pbp2a was obtained from C1/C2 3D masked local refinement of 52403 particles at the resolution of 8.1 Å (phase randomized). The left panel shows the Fourier Shell Correlation FSC (0.143) curves. **B)** 2D class averages obtained from 2D classification of FloA-NfeD dimers. The different 2D classes are presented in the top row. The center row are views of the 2D reprojections of the obtained cryo-EM map whereas the atomic model (Alphafold) is presented in the bottom row. 2D class averages concurring with the 2D reprojections of the cryo-EM map shows the accuracy of data processing.

Supplementary Figure 10

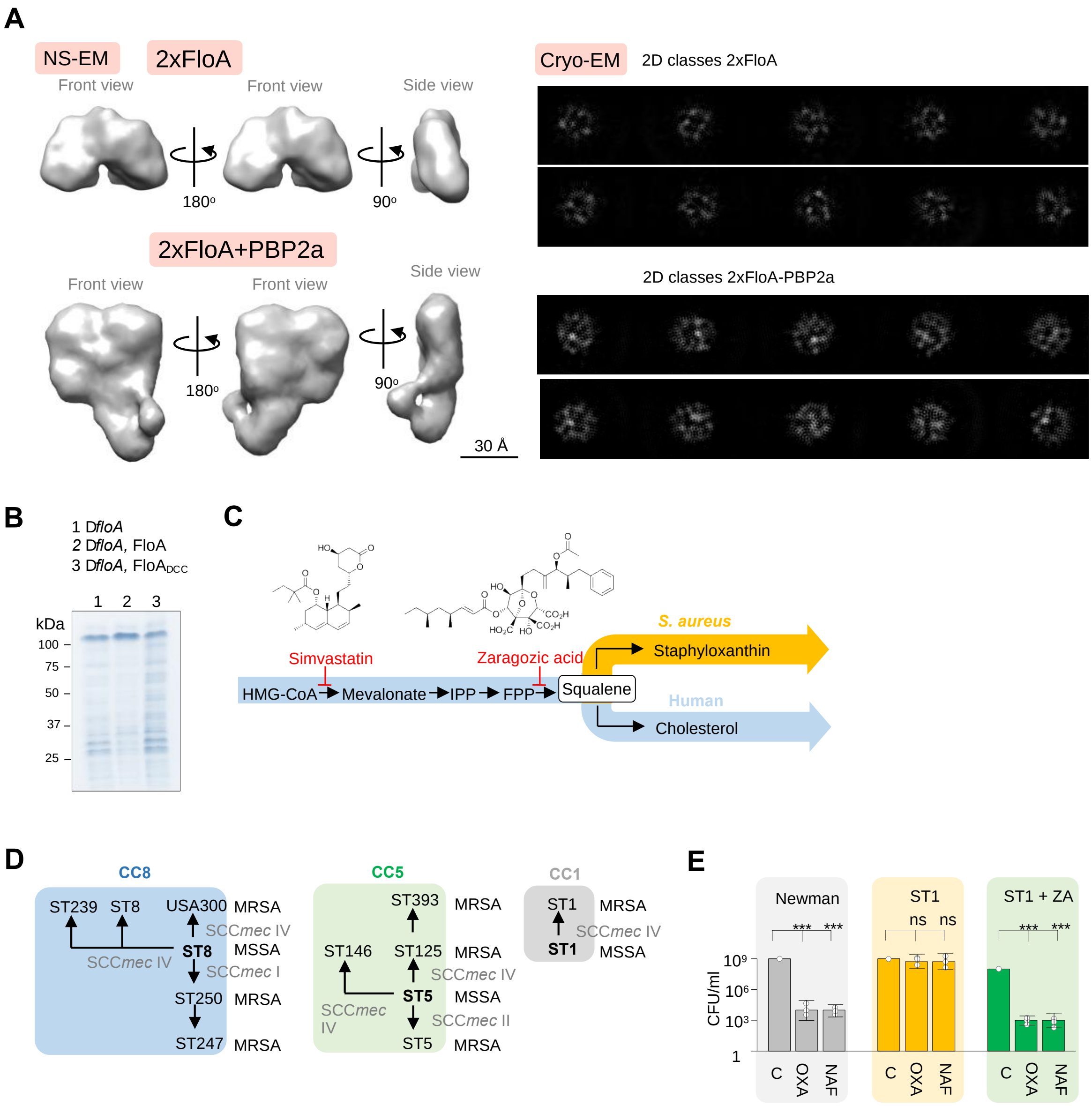

Supplementary Figure 10. FMM disruption leads to the accumulation of unfolded proteins in multi-drug resistant MRSA clinical isolates. A) The left panel shows different views of 3D maps of FloA dimers in their apo state (upper row) or bound to unfolded PBP2a (bottom row) obtained by negative staining EM. The right panel shows 2D class averages obtained from 2D classification of FloA dimers (apo or loaded with PBP2a) obtained by cryo-EM. **B)** SDS-PAGE of insoluble membrane proteins from Δ*floA*, a mutant complemented with a WT variant of *floA* and a Δ*floA* mutant complemented with a *floA*$_{\Delta CC}$ variant with the CC-LCR region deleted. The *floA*$_{\Delta CC}$ variant did not complement the mutant and showed a higher amount of insoluble

membrane proteins than the WT-complemented strain. **C)** Scheme of the mevalonate pathway and its bifurcation to produce staphyloxanthin-related lipids in *S. aureus* (yellow arrow) or cholesterol in humans (blue arrow). Zaragozic acid (ZA) is a competitive inhibitor in both routes, acting downstream of farnesyl pyrophosphate (FPP) formation. Statins also inhibit both routes, as they inhibit the enzyme HMG-CoA reductase. **D)** Evolutionary origins and patterns of descent multilocus sequence type (ST) strains within the clonal complexes (CC) CC8, CC5 and CC1. **E)** Effect on MRSA resistance to several β-lactams using untreated or ZA-treated cultures. Statistical differences were measured by ANOVA with Tukey's test for multiple comparison, *** $p < 0.001$. Data are shown as mean ± SD of three independent experiments ($n = 3$).
