# Supplementary material for: Flotillin-mediated stabilization of unfolded proteins in bacterial membrane microdomains
Source: Nat Commun. 2024 Jul 3;15:5583. doi: 10.1038/s41467-024-49951-1 (PMC11222466; doi:10.1038/s41467-024-49951-1)
Supplement: Supplementary file 2 — Reporting Summary [file 41467_2024_49951_MOESM2_ESM.pdf]

nature portfolio

Corresponding author(s): Daniel Lopez

Last updated by author(s): Jun 16, 2024

Reporting Summary

Nature Portfolio wishes to improve the reproducibility of the work that we publish. This form provides structure for consistency and transparency in reporting. For further information on Nature Portfolio policies, see our Editorial Policies and the Editorial Policy Checklist.

Statistics

For all statistical analyses, confirm that the following items are present in the figure legend, table legend, main text, or Methods section.

n/a	Confirmed	
☐	☒	The exact sample size (*n*) for each experimental group/condition, given as a discrete number and unit of measurement
☐	☒	A statement on whether measurements were taken from distinct samples or whether the same sample was measured repeatedly
☐	☒	The statistical test(s) used AND whether they are one- or two-sided<br>*Only common tests should be described solely by name; describe more complex techniques in the Methods section.*
☒	☐	A description of all covariates tested
☒	☐	A description of any assumptions or corrections, such as tests of normality and adjustment for multiple comparisons
☐	☒	A full description of the statistical parameters including central tendency (e.g. means) or other basic estimates (e.g. regression coefficient) AND variation (e.g. standard deviation) or associated estimates of uncertainty (e.g. confidence intervals)
☐	☒	For null hypothesis testing, the test statistic (e.g. *F*, *t*, *r*) with confidence intervals, effect sizes, degrees of freedom and *P* value noted<br>*Give P values as exact values whenever suitable.*
☒	☐	For Bayesian analysis, information on the choice of priors and Markov chain Monte Carlo settings
☒	☐	For hierarchical and complex designs, identification of the appropriate level for tests and full reporting of outcomes
☒	☐	Estimates of effect sizes (e.g. Cohen's *d*, Pearson's *r*), indicating how they were calculated

Our web collection on statistics for biologists contains articles on many of the points above.

Software and code

Policy information about availability of computer code

Data collection	Size exclusion chromatography GE AKTA Pure<br>Fluorescence microscope Leica DMI6000B<br>NanoLC-MS/MS Thermo Orbitrap Fusion EASY-Spray ion source and EASY-nLC 1000<br>Cryo Electron Microscopes<br>Thermo Talos Arctica Cryo-TEM 200 kV<br>Titan Krios Cryo-TEM 300 kV<br>Electron Microscope JEOL JEM 1400Flash

Data analysis

ChimeraX UCSF https://www.rbvi.ucsf.edu/chimerax
LAS Leica http://www.leica-microsystems.com/products/microscope-software/
Digital Micrograph Gatan https://www.gatan.com/products/tem-analysis/gatan-microscopy-suite-software
IMODFit Chacon Lab https://chaconlab.org/downloads/alphaindex/i
GIMP GNOME Foundation https://gitlab.gnome.org/GNOME/gimp/-/releases/GIMP_2_10_28
Scipion CSIC http://scipion.i2pc.es
Alphafold2 DeepMind https://colab.research.google.com/github/sokrypton/ColabFold/blob/main/beta/AlphaFold2_advanced.ipynb
Vectary Vectary https://www.vectary.com

For manuscripts utilizing custom algorithms or software that are central to the research but not yet described in published literature, software must be made available to editors and reviewers. We strongly encourage code deposition in a community repository (e.g. GitHub). See the Nature Portfolio guidelines for submitting code & software for further information.

Data

Policy information about availability of data

All manuscripts must include a data availability statement. This statement should provide the following information, where applicable:

- Accession codes, unique identifiers, or web links for publicly available datasets
- A description of any restrictions on data availability
- For clinical datasets or third party data, please ensure that the statement adheres to our policy

For proteomic analyses, the raw mass spectrometry data were deposited in the PRIDE repository under the dataset identifiers PDX00654 and PXD041057.
The cryoEM 3D maps have been deposited in EMDB: 1XFloA EMD-17217;
1XFloA-NfeD EMD-17222; 2XFloA-NfeD EMD-17231; 2XFloA-NfeD+PBP2a EMD-17233

Research involving human participants, their data, or biological material

Policy information about studies with human participants or human data. See also policy information about sex, gender (identity/presentation), and sexual orientation and race, ethnicity and racism.

Reporting on sex and gender

Use the terms sex (biological attribute) and gender (shaped by social and cultural circumstances) carefully in order to avoid confusing both terms. Indicate if findings apply to only one sex or gender; describe whether sex and gender were considered in study design; whether sex and/or gender was determined based on self-reporting or assigned and methods used.
Provide in the source data disaggregated sex and gender data, where this information has been collected, and if consent has been obtained for sharing of individual-level data; provide overall numbers in this Reporting Summary. Please state if this information has not been collected.
Report sex- and gender-based analyses where performed, justify reasons for lack of sex- and gender-based analysis.

Reporting on race, ethnicity, or other socially relevant groupings

Please specify the socially constructed or socially relevant categorization variable(s) used in your manuscript and explain why they were used. Please note that such variables should not be used as proxies for other socially constructed/relevant variables (for example, race or ethnicity should not be used as a proxy for socioeconomic status).
Provide clear definitions of the relevant terms used, how they were provided (by the participants/respondents, the researchers, or third parties), and the method(s) used to classify people into the different categories (e.g. self-report, census or administrative data, social media data, etc.)
Please provide details about how you controlled for confounding variables in your analyses.

Population characteristics

Describe the covariate-relevant population characteristics of the human research participants (e.g. age, genotypic information, past and current diagnosis and treatment categories). If you filled out the behavioural & social sciences study design questions and have nothing to add here, write "See above."

Recruitment

Describe how participants were recruited. Outline any potential self-selection bias or other biases that may be present and how these are likely to impact results.

Ethics oversight

Identify the organization(s) that approved the study protocol.

Note that full information on the approval of the study protocol must also be provided in the manuscript.

Field-specific reporting

Please select the one below that is the best fit for your research. If you are not sure, read the appropriate sections before making your selection.

☒ Life sciences ☐ Behavioural & social sciences ☐ Ecological, evolutionary & environmental sciences

For a reference copy of the document with all sections, see nature.com/documents/nr-reporting-summary-flat.pdf

Life sciences study design

All studies must disclose on these points even when the disclosure is negative.

Sample size	No statistical methods were used to predetermine sample size. The sample size was chosen to include at least 3 biologically independent experiments. Sample size was based on standard sample sizes from our past experiments and similarly to what is described for similar experiments in published articles (Koch et al., Cell 2014, 158:1060 and García-Fernández and Koch et al., Cell 2017, 171:1354)
Data exclusions	No data were excluded from the analyses
Replication	Experiments were independently performed at least 3 times and all attempts of replication were successful.
Randomization	Animals were randomly assigned to experimental groups. For in vitro experiments, cultured cells were uniformly plated, with random allocation of infection.
Blinding	The researchers performing proteomic analyses were blinded to strain selection. For the remaining experiments, blinding was not applicable given the nature of the study. All critical experiments were repeated independently by at least two researchers.

Reporting for specific materials, systems and methods

We require information from authors about some types of materials, experimental systems and methods used in many studies. Here, indicate whether each material, system or method listed is relevant to your study. If you are not sure if a list item applies to your research, read the appropriate section before selecting a response.

Materials & experimental systems

n/a	Involved in the study	
☐	☒	Antibodies
☐	☒	Eukaryotic cell lines
☒	☐	Palaeontology and archaeology
☐	☒	Animals and other organisms
☒	☐	Clinical data
☒	☐	Dual use research of concern
☒	☐	Plants

Methods

n/a	Involved in the study	
☒	☐	ChIP-seq
☒	☐	Flow cytometry
☒	☐	MRI-based neuroimaging

Antibodies

Antibodies used	anti-FloA chicken Davids (Garcia-Fernandez et al., 2017)<br>anti-FLAG rabbit Sigma Cat#F7425<br>anti-HIS rabbit Rockland Cat#200-301-B90<br>anti-PBP2a rabbit RayBiotech Cat#130-10073-100<br>anti-chicken HRP-conjugated secondary Thermo Cat#A1654<br>anti-rabbit HRP-conjugated secondary BioRad Cat#1706515
Validation	All antibodies were validated by the respective manufacturers. Validation in this study were performed by comparing labelled and unlabelled strains.

Eukaryotic cell lines

Policy information about cell lines and Sex and Gender in Research

Cell line source(s)	THP1 Macrophages ATCC TIB-202
Authentication	None of the cell lines was authenticated.
Mycoplasma contamination	All cells were tested and negative for Mycoplasma contamination
Commonly misidentified lines<br>(See ICLAC register)	No commonly misidentified cell lines were used in this study.

Animals and other research organisms

Policy information about studies involving animals; ARRIVE guidelines recommended for reporting animal research, and Sex and Gender in Research

Laboratory animals	Inbred female mice BALB/c weighing 20 g to 24 g purchased from Charles River Laboratories.
Wild animals	No wild animals were involved.
Reporting on sex	Sex was not considered in this study
Field-collected samples	No field-collected samples were involved.
Ethics oversight	Committee on Ethics in Animal Experiments of the Government of Madrid with the approval code PROEX009-18

Note that full information on the approval of the study protocol must also be provided in the manuscript.
