# Supplementary material for: Flotillin-mediated stabilization of unfolded proteins in bacterial membrane microdomains
Source: Nat Commun. 2024 Jul 3;15:5583. doi: 10.1038/s41467-024-49951-1 (PMC11222466; doi:10.1038/s41467-024-49951-1)
Supplement: Supplementary file 4 — Description of Additional Supplementary Files [file 41467_2024_49951_MOESM4_ESM.pdf]

Description of Additional Supplementary Files

Supplementary Movie 1. AlphaFold2 prediction of FloA and NfeD. Movie of FloA (upper panel) and NfeD (bottom panel) structural prediction according to AlphaFold2. The regions of the proteins are colored according to the per-residue confidence score (pLDDT) between 0 and 100.

Supplementary Movie 2. Cryo-EM map of FloA monomer (1XFloA). Movie of FloA monomer (1XFloA) cryo-EM volume and the fitted atomic model predicted by AlphaFold2. FloA is represented in dark grey.

Supplementary Movie 3. Cryo-EM map of FloA-NfeD monomer (1XFloA-NfeD). Movie of FloA-NfeD monomer (1XFloA-NfeD) cryo-EM volume and the fitted atomic model predicted by AlphaFold2. FloA is represented in dark grey and the OBL of NfeD is represented in blue.

Supplementary Movie 4. Cryo-EM map of FloA-NfeD dimer (2XFloA-NfeD) in its apo version. Movie of the apo FloA-NfeD dimer (2XFloA-NfeD) cryo-EM volume and the fitted atomic model predicted by AlphaFold2. The two FloA of the dimer are represented in dark grey. The OBL of NfeD are represented in blue.

Supplementary Movie 5. Cryo-EM map of FloA-NfeD dimer (2XFloA-NfeD) in its loaded version with PBP2a. Movie of the PBP2a-loaded FloA-NfeD dimer (2XFloA-NfeD + PBP2a) cryo-EM volume and the fitted atomic model predicted by Alphafold2. The two FloA of the dimer are represented in dark grey. The OBL of NfeD are represented in blue. The PBP2a volume is embraced by the two FloA tentacles and is represented in green.

Supplementary Data 1. List of NfeD proteins from different bacterial species used to generate the phylogenetic classification shown in Extended Data Fig S1Aii.

Supplementary Data 2. Classification of cryo-EM analyses performed in this study.

Supplementary Data 3. Functional classification of the proteins detected in abundance in DRM vs DSM by unsupervised hierarchical clustering. Functional classification was performed according to TIGRFAMM classification guidelines.

Supplementary Data 4. Identification of the proteins detected in thermal proteome profiling. The table compares protein abundance between the different sampling points (steps) of the same strain as well as the protein abundance between different strains (WT vs. Δ*floA*) of the same sampling point.

Supplementary Data 5. Multilocus sequence typing (MLST) classification of the multi-drug resistant MRSA clinical isolates used in this study.

Supplementary Data 6. Detection of antibiotic-resistant genes in the multi-drug resistant MRSA clinical isolates used in this study. *A7J11_00087*, bifunctional aminoglycoside N-acetyltransferase; *aad1*, aminoglycoside adenyltransferase; *ant(6)-1a*, aminoglycoside O-nucleotidyltransferases; *aph(3')-IIIa*, aminoglycoside 3'-phosphotransferase; *bla1*, β-lactamase-1; *blaPC1*, PC1 β-lactamase; *blaR1*, β-lactamase regulatory protein R1; *blaZ*, β-lactamase; *bleO*, bleomycin resistance; *catA*, chloramphenicol acetyltransferase; *dfrC*, dihydrofolate

reductase (trimethoprim resistance); *erm(c)*, erythromycin resistance; *fosD*, metallothiol transferase (fosfomycin resistance); *lnu(B)*, lincosamide nucleotidyltransferase; *mecA*, methicillin resistance penicillin-binding protein; *mecR1*, methicillin resistance R1 protein; *mphC*, macrolide 2'-phosphotransferase; *msr(A)*, macrolide/streptogramin resistance; *sat4*, strepthothricin acetyltransferase; *tet(38)*, tetracycline resistance; *tetC*, tetracycline resistance; tet(k), tetracycline resistance.

Supplementary Data 7. List of strains used in this study.

Supplementary Data 8. List of primers used in this study.
