# Supplementary material for: Flotillin-mediated stabilization of unfolded proteins in bacterial membrane microdomains
Source: Nat Commun. 2024 Jul 3;15:5583. doi: 10.1038/s41467-024-49951-1 (PMC11222466; doi:10.1038/s41467-024-49951-1)
Supplement: Supplementary file 11 — Source data [file 41467_2024_49951_MOESM11_ESM.zip › Source Data.pdf]

Figure 2A

ii

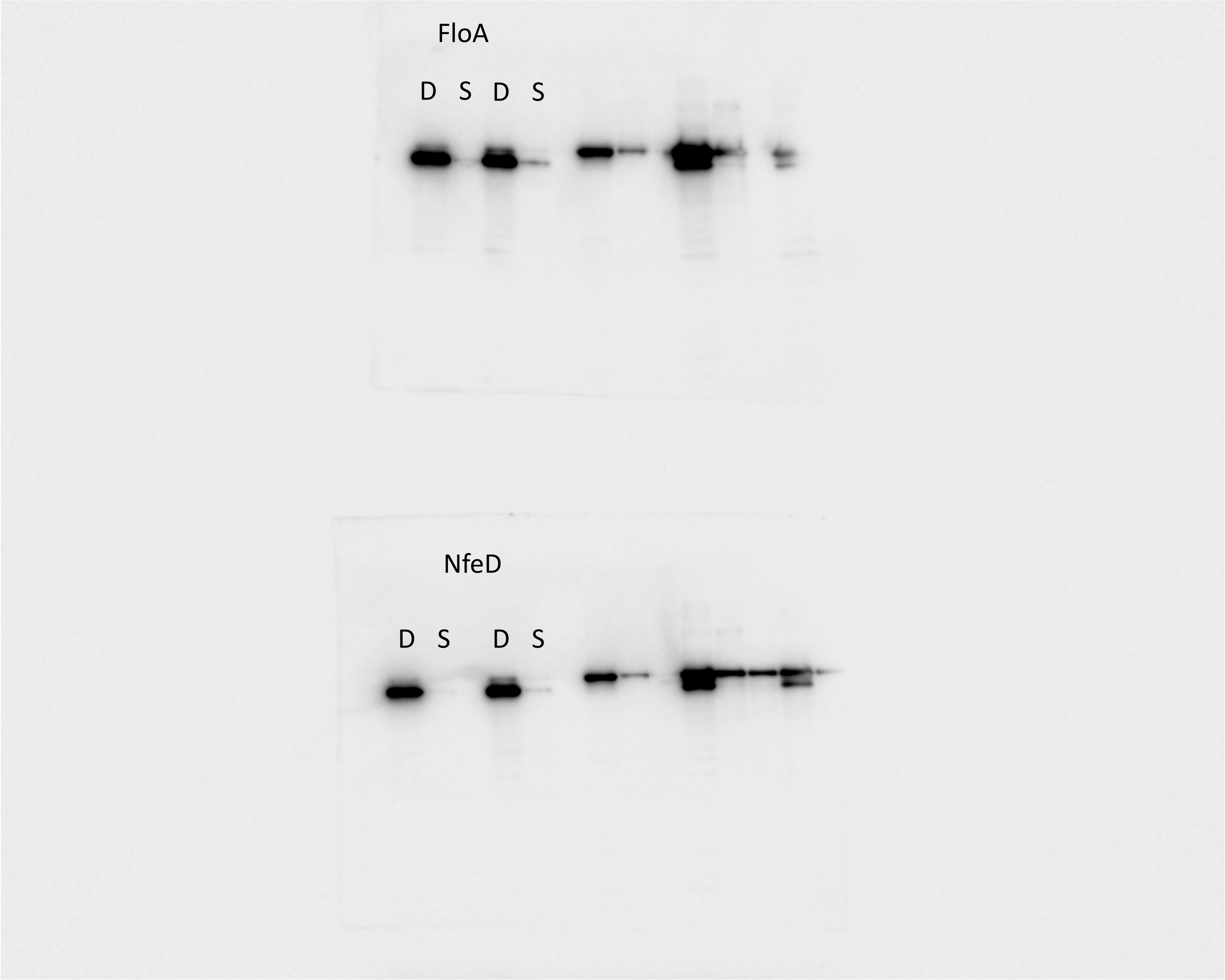

Figure 2B

i

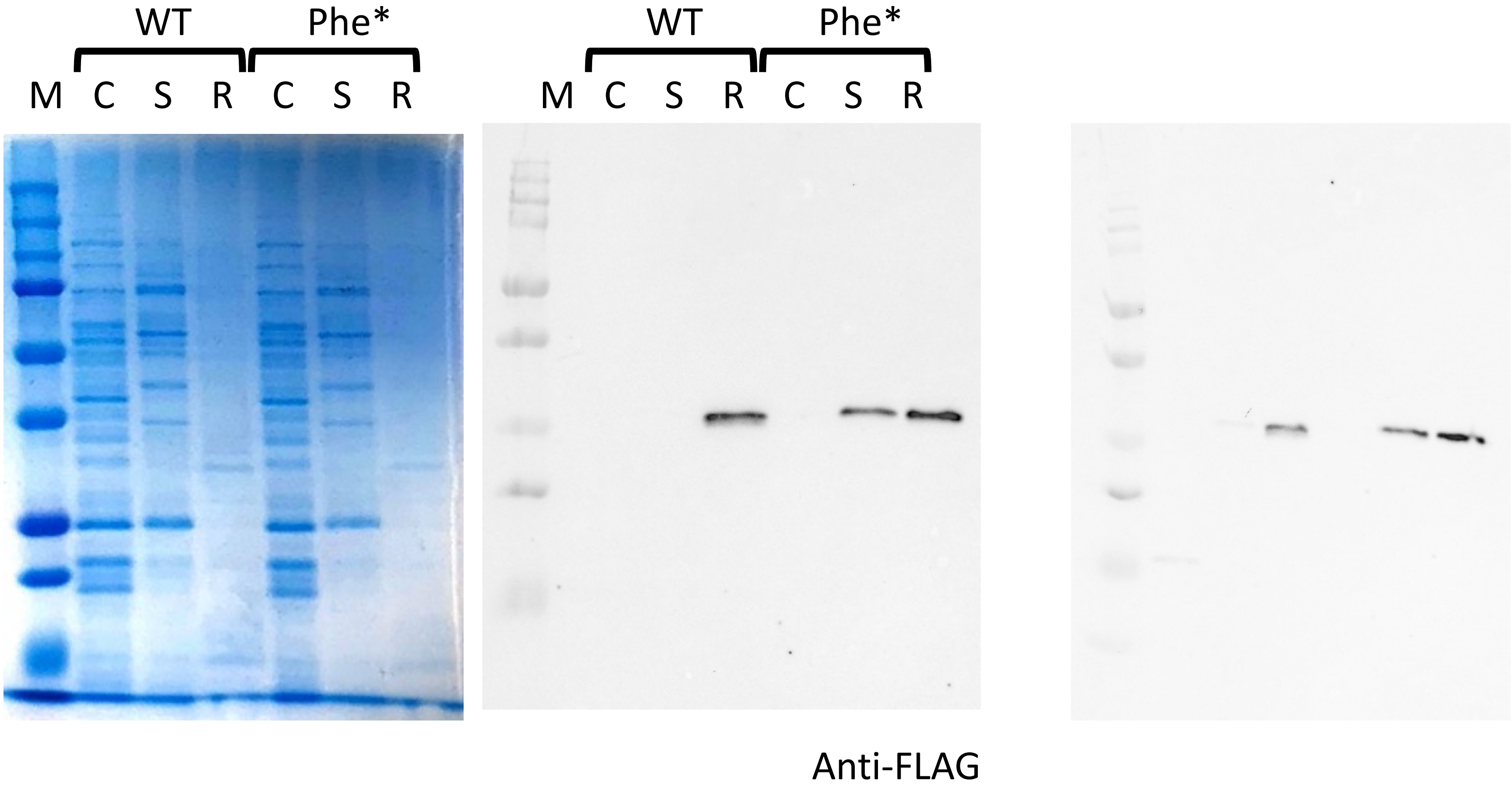

Figure 2C

ii

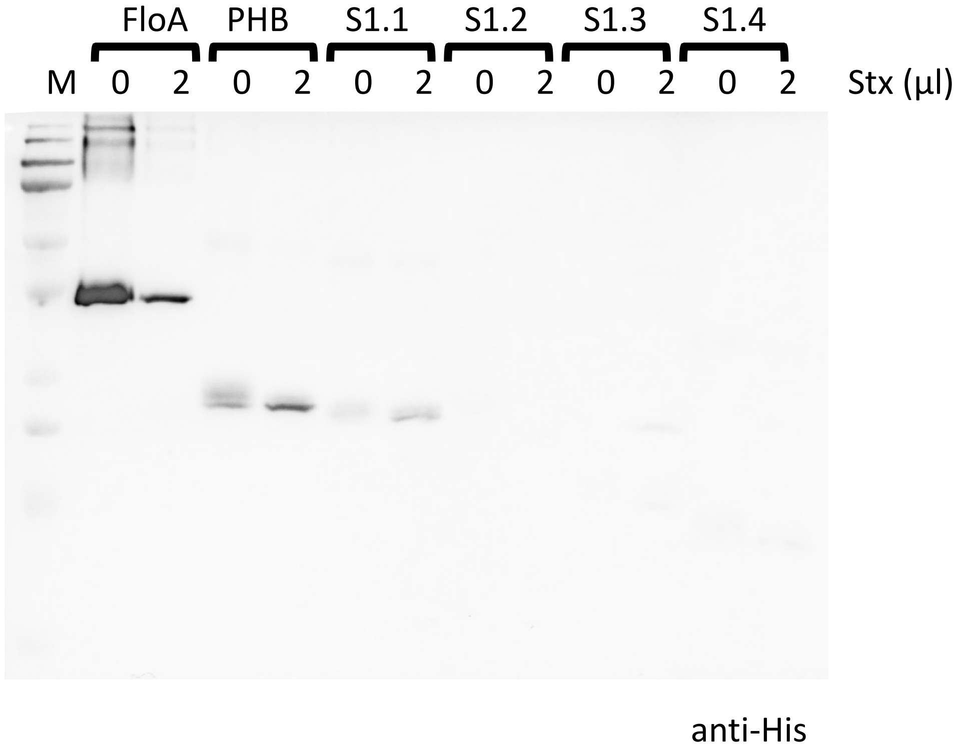

iii

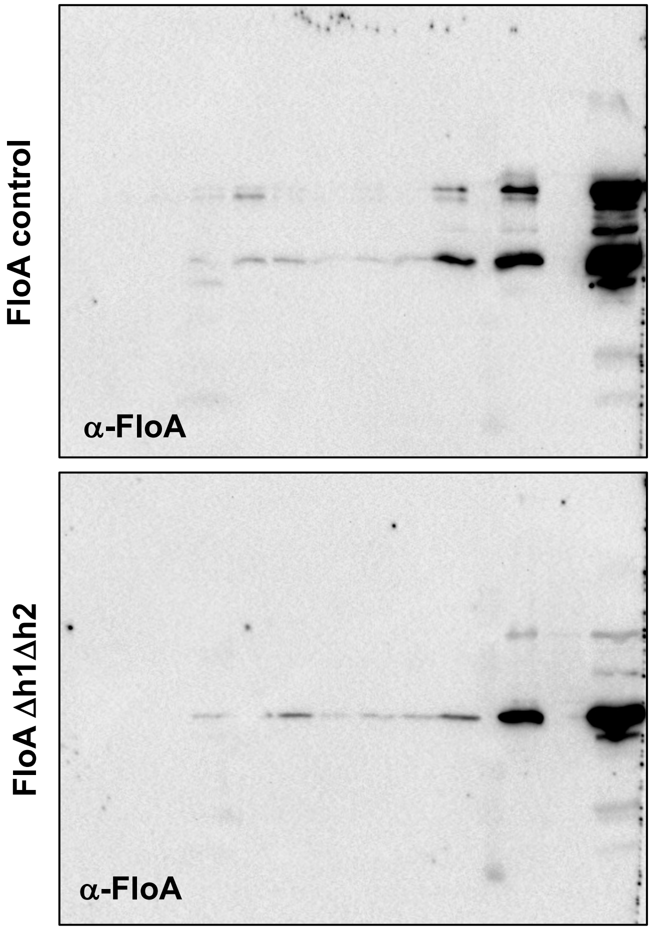

Figure 2D

i

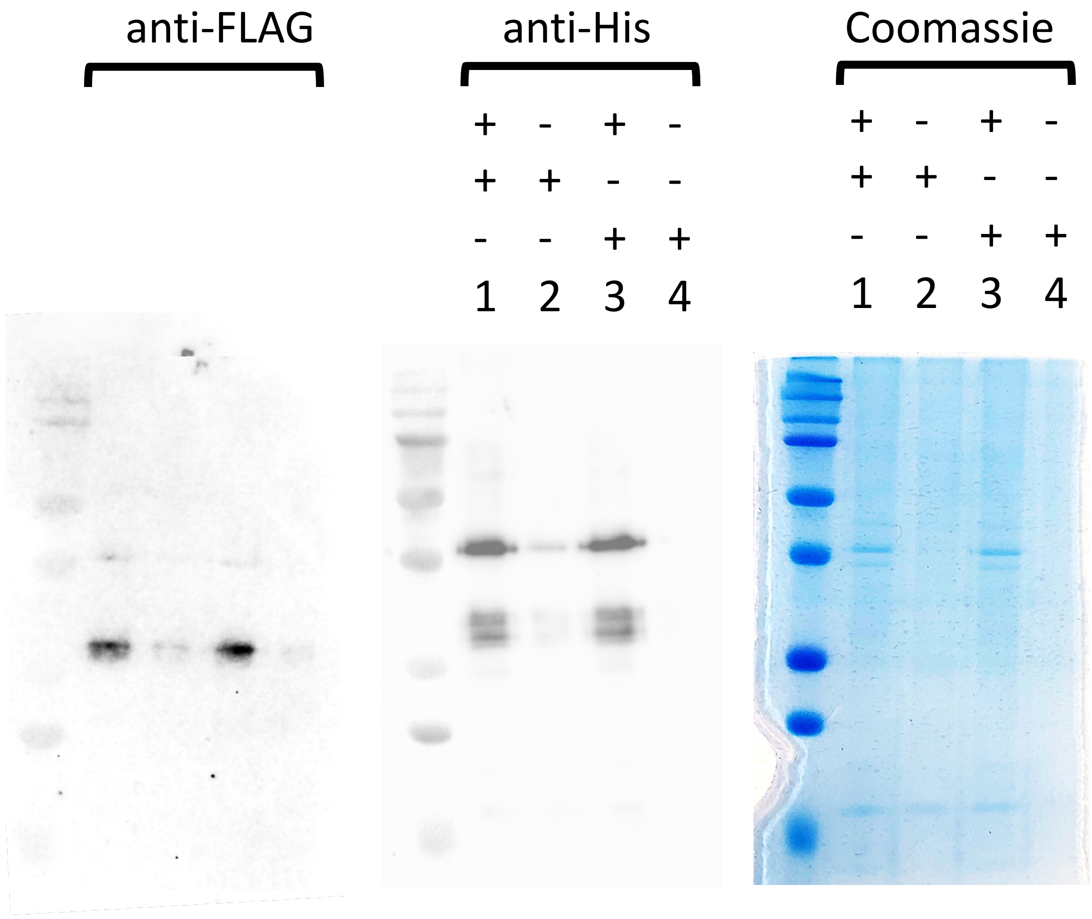

FloA = 38 kDa
NfeD = 26 kDa

Figure 4B

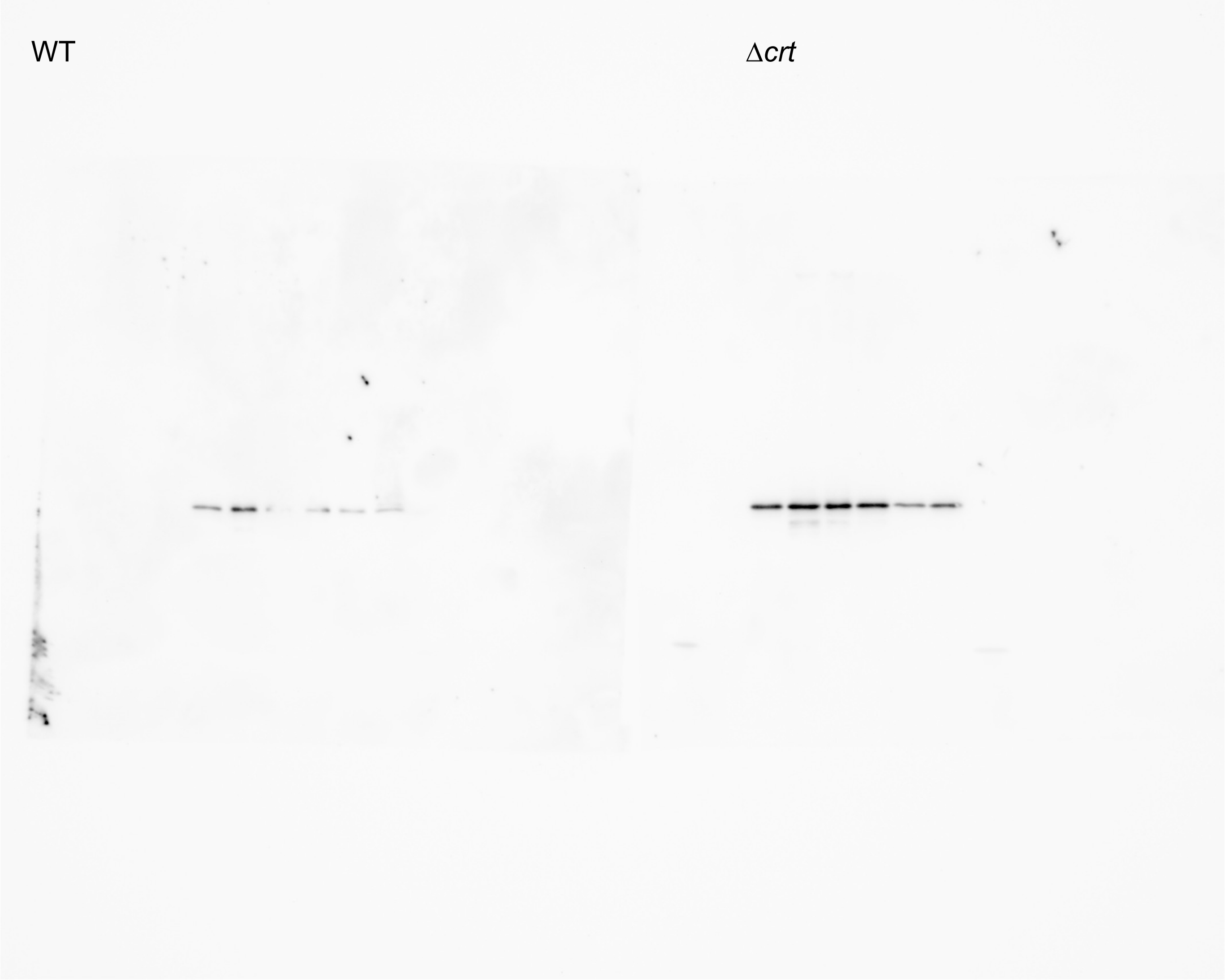

Figure 5A

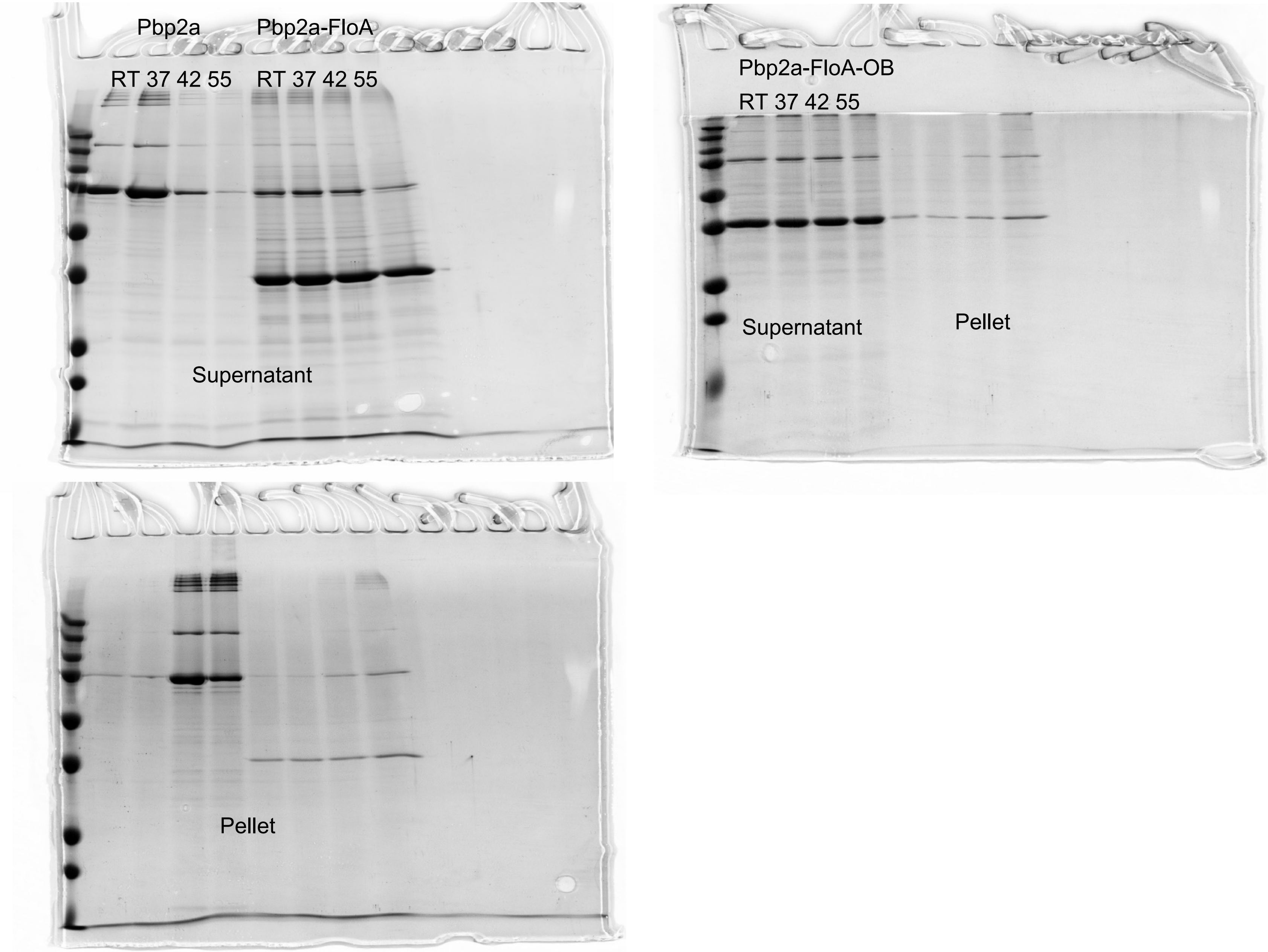
Pbp2a
Pbp2a-FloA
RT 37 42 55
RT 37 42 55
Supernatant
Pbp2a-FloA-OB
RT 37 42 55
Supernatant
Pellet
Pellet

Figure 6D

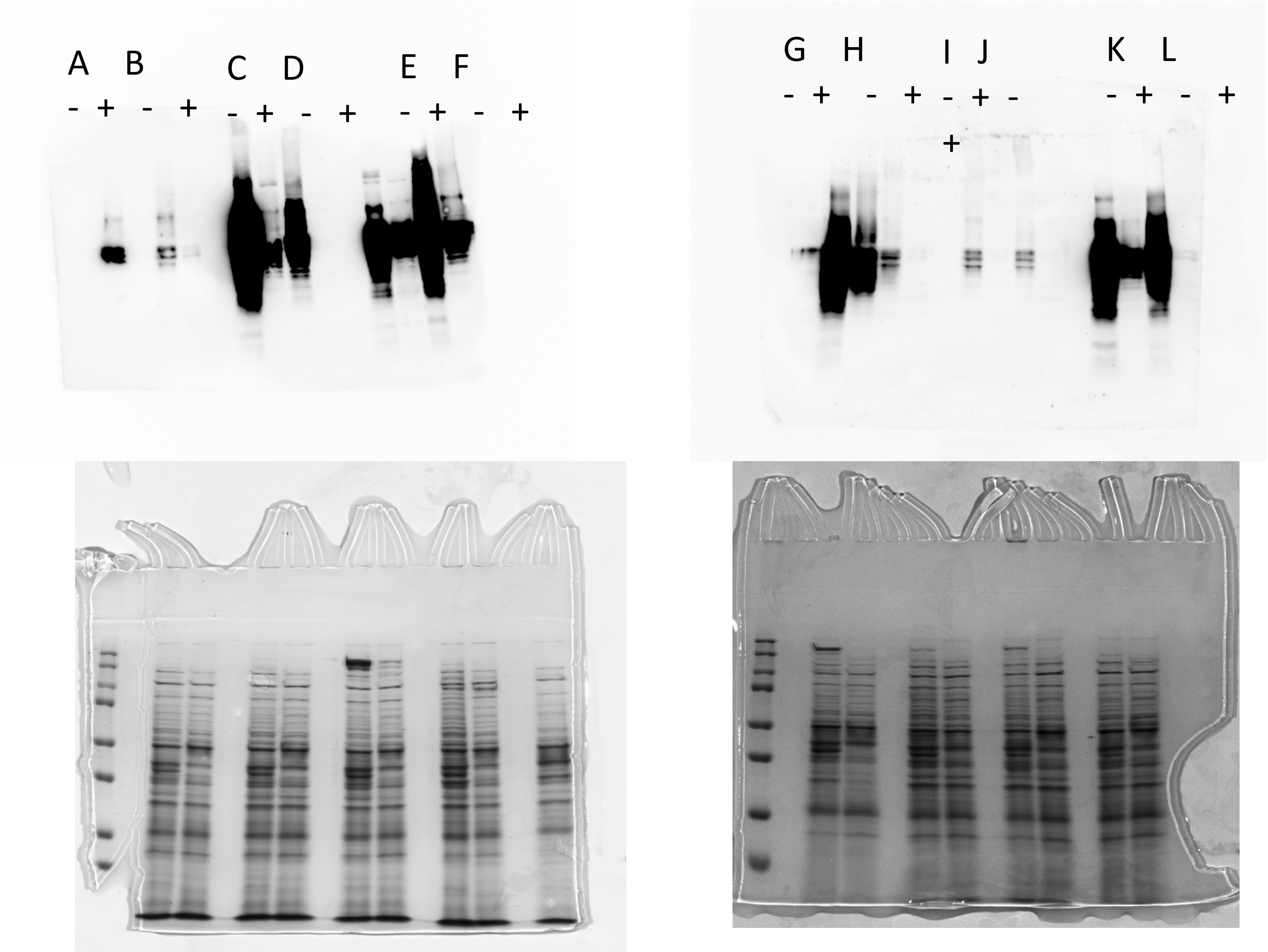

Figure 6E

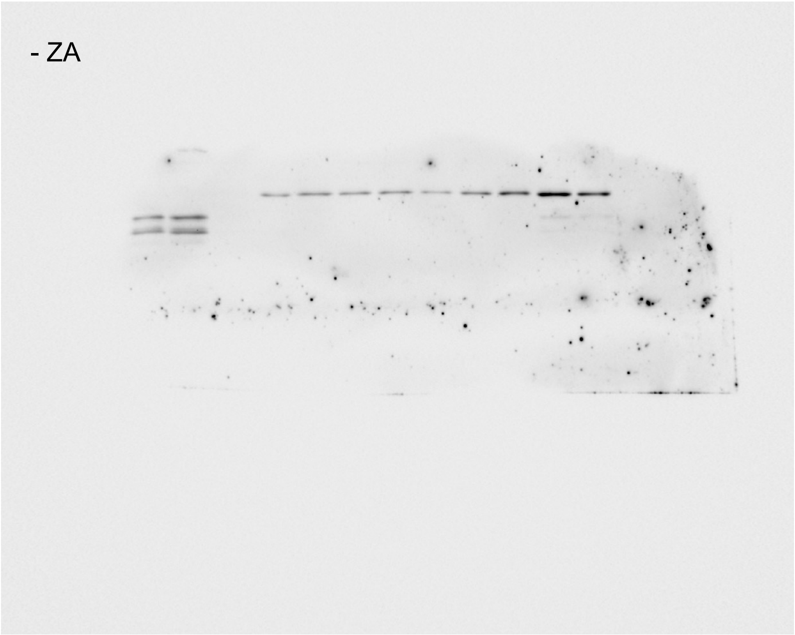

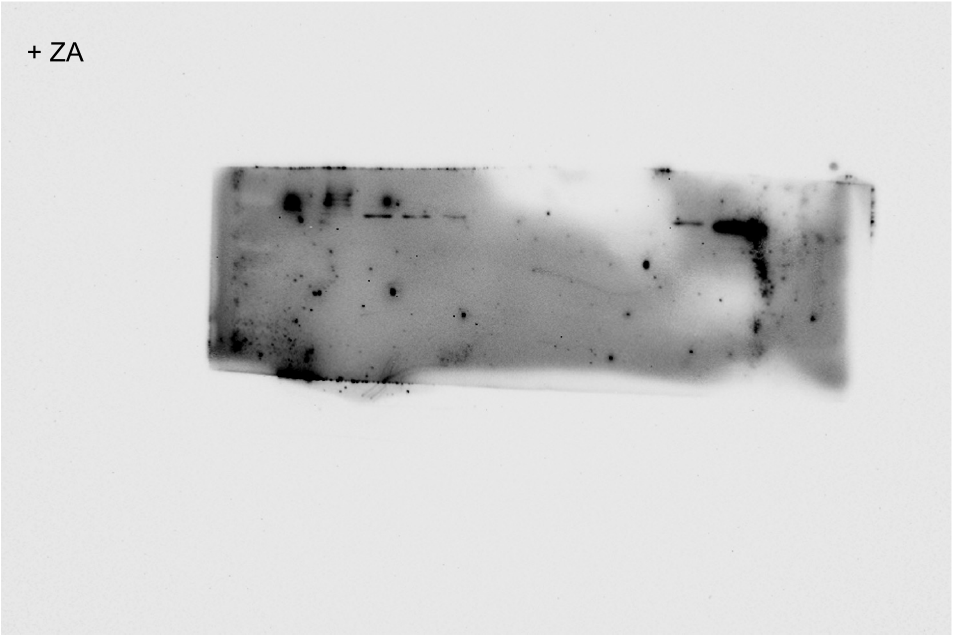

FloA (α-FloA)
